# Ectonucleoside Triphosphate Diphosphohydrolase-1/CD39 Affects the Response to ADP of Female Rat Platelets

**DOI:** 10.3389/fphar.2019.01689

**Published:** 2020-01-31

**Authors:** Elisabetta Caiazzo, Rossella Bilancia, Antonietta Rossi, Armando Ialenti, Carla Cicala

**Affiliations:** Department of Pharmacy, School of Medicine and Surgery, University of Naples Federico II, Naples, Italy

**Keywords:** adenosine diphosphate, aggregation, CD39, platelet, thrombosis, inflammation, rat, sex

## Abstract

There is evidence that an imbalance of extracellular purine levels may be associated with increased cardiovascular risk. Platelets play a pivotal role in vascular homeostasis and thrombosis and are important source of purine nucleotides and nucleosides. Hydrolysis of nucleotides ATP and ADP is regulated by two ectonucleotidases, triphosphate diphosphohydrolase-1 (NTPDase-1/CD39) and ecto-5’-nucleotidase (ecto-5’-NT/CD73). CD39 enzyme is expressed on the endothelium, circulating blood cells, and smooth muscle cells; there is evidence that changes in CD39 expression and activity affects the potential thrombogenic of a tissue. Gender difference in the cardiovascular risk has been extensively observed; however, while the age-dependent difference in the prevalence of cardiovascular events between men and women has been attributed to the loss of the protective effect of estrogens in the postmenopausal period, the physiological mechanism behind gender disparity is still unclear. Here, we evaluated comparatively male and female rat platelet reactivity and considered the possible role of CD39 at the basis of difference observed. We found a reduced *in vitro* response to ADP (1–30 µM) of female compared to male platelets, associated to increased platelet CD39 expression and activity. Platelet response to ADP was strongly increased by incubation (10 min) with the CD39 inhibitor, ARL67156 (100 µM), while male platelet response was unaffected. Rat treatment with clopidogrel (30 mg/kg, *per os*) inhibited ex vivo platelet aggregation. Bleeding time was prolonged in female compared to male. Taken together, our results suggest that platelet ATPase and ADPase activity might be a reliable predictor of platelet reactivity.

## Introduction

An increased cardiovascular risk may be associated with an imbalance of extracellular purine levels (Ajjan and Grant, 2006; Jalkanen et al., 2015).

Circulating blood platelets are an important source of purine nucleotides and nucleosides. In particular, in a damaged tissue, adenosine diphosphate (ADP) released from activated platelets contributes to stimulate other platelets and to consolidate aggregates, by signaling through P2Y_1_ and P2Y_12_ receptors (Burnstock, 2017).

Hydrolysis of nucleotides adenosine triphosphate (ATP) and ADP is regulated mainly by membrane bound nucleoside triphosphate diphosphohydrolase-1 (NTPDase-1) also known as CD39. A related ecto-5’-nucleotidase (ecto-5’-NT), CD73, hydrolyzes adenosine monophosphate (AMP) into adenosine (Antonioli et al., 2013). CD39 enzyme is expressed on the endothelium, circulating blood cells, and smooth muscle cells, and it is able to hydrolyze almost directly ATP to AMP (Pulte et al., 2007; Behdad et al., 2009). Besides CD39, other cell surface located NTPDase subtypes have been identified, NTPDase 2, 3, and 8. While the hydrolyzing activity of NTPDase 3 and 8 leads to the simultaneous presence of ATP and ADP, NTPDase-2 preferentially hydrolyze ATP, leading to ADP accumulation (Robson et al., 2006).

There is evidence that in an inflammatory environment, the loss of CD39 activity from activated endothelium sustains platelet aggregation and thrombogenesis (Atkinson et al., 2006). On the other hand, within a damaged tissue the increased expression of CD39 on inflammatory cells, working in tandem with CD73, might cause inhibition of platelet activation by increasing extracellular adenosine levels (Johnston-Cox et al., 2011). Hence, changes in CD39 expression and activity affect the thrombogenic potential of a tissue (Covarrubias et al., 2016; Anyanwu et al., 2019; Yadav et al., 2019). Consistently, it has been demonstrated that a reduction of CD39 expression and activity is associated with thrombotic disorders coming along with different pathological conditions (Deaglio and Robson, 2011; Samudra et al., 2018).

Sex has been emerging as an important variable in several physiopathological conditions (Kim et al., 2010; Rossi et al., 2019); gender differences in the thrombotic and bleeding risks have been well described (Breet et al., 2011). There is evidence of higher prevalence of cardiovascular diseases (CVDs) in younger men than in women as well as of dramatic increase in cardiovascular risk in postmenopausal women (Regitz-Zagrosek, 2006; Kim and Monon, 2009). While the age-dependent difference in the prevalence of cardiovascular events between male and female has been attributed to the loss of the protective effect of estrogens in the postmenopausal period, the physiological mechanism behind gender disparity is still unclear (Iorga et al., 2017). Moreover, gender difference in the effect of cardiovascular drugs has also been reported (Stolarz and Rusch, 2015).

The antiplatelet therapy represents the cornerstone in the prevention of cardiovascular risk (Patrono et al., 2017). Change in platelet reactivity plays a preeminent role in thrombus formation and may affect the response to therapy. Here, we have evaluated comparatively platelet functionality in male and female rats and the possible link to CD39 enzyme.

## Materials and Methods

### Animals

All experiments were performed on male and female Wistar rats (8 weeks of age, Charles River, Calco, Italy). The animals were maintained at a room temperature of 22 ± 2° on a 12-h/12 h light/dark cycle and were housed in a specific pathogen-free environment and fed standard rodent chow and water ad libitum. All procedures were carried out in accordance with the principles of the Basel Declaration and recommendation according to the European (n.63/2010/UE) and to Italian (DL26/2014) regulations on the protection of animals used for experimental and other scientific purposes. The protocol was approved by the Italian Ministry of Health (according to DL 26/2014; protocol project no. 459/2019-PR).

### Preparation of Platelet-Rich Plasma and Platelet Aggregation

Blood was withdrawn by cardiac puncture from rats slightly anesthetized with enflurane, and anticoagulated with 3.8% (w/v) trisodium citrate (1:9 v/v). Platelet-rich plasma (PRP) and platelet-poor plasma (PPP) were prepared as previously described (Cicala et al., 2007, Caiazzo et al., 2016). Briefly, PRP was obtained by centrifugation at 200 × g for 15 min at 25°C. PPP was prepared from the remaining blood by centrifugation at 600 × g for 15 min at 25°C. Platelet count in PRP was performed by a cell counter (Beckman Coulter s.r.l., Milano, Italy) and adjusted to 3 × 10^5^ platelets/μl with autologous PPP. Platelet aggregation was monitored by a lumiaggregometer (Chrono-Log, Corporation, Mod.490, USA) according to Born and Cross (1963) by measuring changes in turbidity of 0.25 ml of re-calcified (CaCl_2_ 1mM) PRP warmed at 37°C and under continuous stirring. A single concentration–response curve (1–30 µM) to ADP was evaluated. The maximum platelet aggregation rate was recorded within 16 min with continuous stirring at 37°C. In some experiments, platelets were also pre-incubated (10 min) with CD39 inhibitor, ARL67156 trisodium salt (100 µM, Tocris Bioscience, Bristol, UK). Platelet aggregation was determined by software AGGRO/LINK (Chrono-Log, Havertown, PA, USA) and evaluated in terms of amplitude (maximum % of aggregation).

### Rat Treatment

Different groups of animals were treated with (+)-clopidogrel hydrogen sulfate (30 mg/kg; Tocris Bioscience, Bristol, UK) or vehicle (0.5% sodium carboxymethyl cellulose) per os (by gastric gavage), 2 h before blood sampling and tail transaction. Dosage of clopidogrel was established on the basis of literature (Ogawa et al., 2009; Xu et al., 2018).

### Bleeding Time

The bleeding time was determined using a cutting-tail rat model. Rats were anesthetized by intraperitoneal injection of a mixture of Zoletil^®^ (30 mg/kg) and xilazine (10 mg/kg). The rat tail was transectioned with a scalpel at a point 3 mm from the tip and immersed in 37°C saline (Elg et al., 1999). The tail was pre-warmed by immersion in saline for 3 min before the cut. Blood flowing from the incision was carefully monitored and bleeding time was recorded as the time between the blood overflowed and the very first moment when the bleeding stopped.

### Western Blot Analysis

Platelet suspension was washed with Phosphate Buffered Saline (PBS) and centrifuged at 600 × g for 15 min at 25°C. The platelet pellet was then lysed in the radioimmunoprecipitation assay (RIPA) buffer with protease inhibitor (Sigma-Aldrich, Italy). Total protein concentration was determined by Bradford assay, using BSA (bovine serum albumin) as standard. Protein samples (50 µg) were separated by 8% Sodium Dodecyl Sulphate - PolyAcrylamide Gel Electrophoresis (SDS-PAGE) and then transferred onto a nitrocellulose membrane using standard procedure. The membranes were saturated by incubation with 5% non-fat dry milk in PBS supplemented with 0.1% Tween-20 (PBS-T) for 1 h at room temperature and primary antibody was incubated overnight at 4°C. Antibodies used on different membranes were: anti-CD39 goat (1:200; Santa Cruz Biotechnology, Italy), anti-cyclooxygenase 1 rabbit (COX-1, 1:1,000; Cell Signaling Technology Inc., USA), anti-CD73 goat (1:200; Santa Cruz Biotechnology, Italy), anti-P2Y_1_ mouse (1:500; Invitrogen Carlsbad, CA), and anti-P2Y_12_ rabbit (1:2,000; Abcam, Cambridge, UK). After washing, membranes were incubated with the appropriated secondary antibody. Protein bands were normalized over the intensity of the housekeeping β-actin. Immunoreactive bands were detected using the enhanced chemiluminescence (ECL) detection kit and Chemidoc Imaging System (Bio-Rad Laboratories Inc.). Densitometry was performed with ImageLab software (Bio-Rad Laboratories Inc.).

### Measurement of Specific ATPase and ADPase Activity

Platelet ATPase and ADPase activity was determined by measuring the concentrations of inorganic phosphate (P*i*) with the Malachite Green assay kit (CliniSciences, Italy), as previously described (Caiazzo et al., 2016). Briefly, platelet lysates (50 μg) were pre-incubated in 200 μl of reaction buffer containing NaCl (10 mM), KCl (5 mM), glucose (60 mM), CaCl_2_ (5 mM), and Tris-HCl (50 mM), pH 7.5, at 37°C for 10 min. The enzyme reaction was started by the addition of ATP or ADP to a final concentration of 1 mM; after 40 min at 37°C, the reaction was stopped by the addition of 200 μl of trichloroacetic acid (TCA). The concentration of P*i* released during the hydrolysis of ATP and ADP was measured using the Malachite Green assay according to the manufacturer’s instructions. To determine specificity, experiments were also performed in the presence of the CD39 inhibitor, ARL67156 trisodium salt (100 µM, Tocris Bioscience, Bristol, UK). For these experiments, samples were incubated with ARL67156 in assay medium for 30 min at 37°C before adding ATP or ADP. To have the net value of P*i* produced following enzymatic reaction, non-specific P*i* released in the presence of ARL67156 in each sample was subtracted from the value obtained following incubation with the substrate. Results were expressed as P*i* released pmol/min/µg protein.

### Statistical Analysis

All results are expressed as mean ± standard error (SE) of N = 5–6. Concentration–response curves were represented by nonlinear regression and analyzed by two-way analysis of variance (ANOVA) followed by Bonferroni’s test. In other cases, two-tailed t test, as appropriated, was used. All statistical analysis was performed using GraphPad, Prism V5.0 (GraphPad software, California, USA). A value of p < 0.05 was considered statistically significant.

## Results

### CD39 Inhibition Increases Female Platelet Aggregation

**Figure 1:**
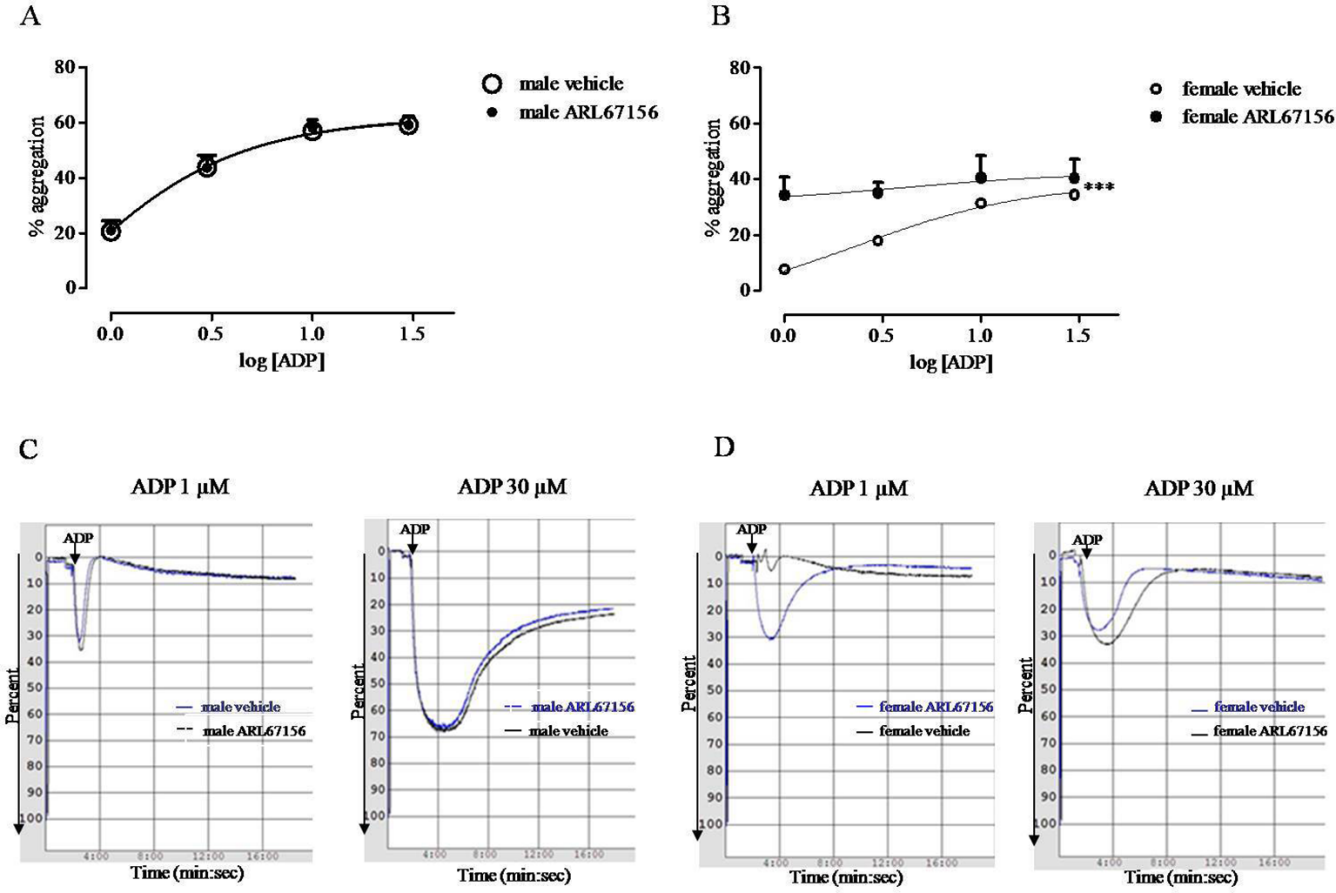
Sex difference in platelet response to ADP (1–30 µM); the *in vitro* effect of CD39 inhibitor (ARL67156) was evaluated. Platelet-rich plasma (PRP) from male **(A)** and from female **(B)** rats was incubated with ARL67156 (100 µM, 10 min) or with the vehicle (distilled water) before a single ADP concentration. Aggregation was monitored over a 16 min period, and then quantified and expressed as a percentage of maximum amplitude **(A, B)**. Curves were analyzed by nonlinear regression. ***p < 0.001, two-way ANOVA, N = 6. Typical records of platelet aggregation showing the effect of ARL67156 are also reported **(C, D)**.

Platelet aggregation in response to ADP (1–30 µM) was significantly reduced in female compared to male rats. Concentration–response curve analysis of platelet aggregation shows that the Emax was 61.88 ± 3.55% for male and 38.40 ± 6.58% for female rats (p< 0.001, n = 6; **Figure 1**). Platelet incubation with CD39 inhibitor, ARL67156 (100 µM, 10 min), caused the increased response to ADP of platelet from female rats but did not have any effect on the response to ADP of platelet from male rats (**Figure 1**).

### Female Platelets Show Increased CD39 Expression

**Figure 2:**
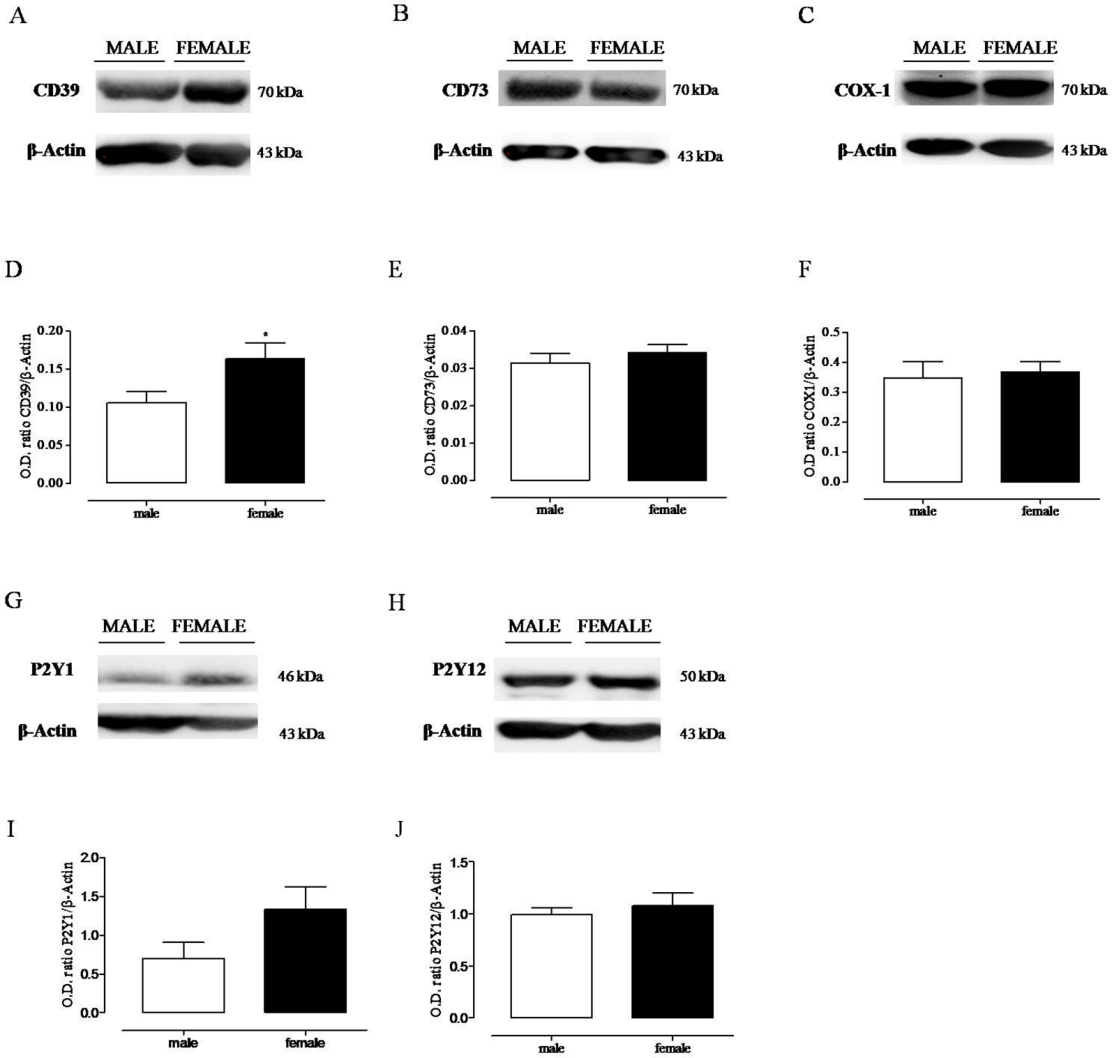
Expression of CD39, CD73, cyclooxygenase-1, P2Y_1_, P2Y_12_ **(A–C, G, H)** evaluated by Western blot analysis in unstimulated platelet lysates from male and female rats. Densitometry of specific bands has been normalized to β-actin expression **(D–F, I, J)**. * p < 0.05 *versus* male, two-tailed t test, N = 6.

On platelet from female rats, the expression of CD39 was significantly increased compared to the expression on platelet from male rats (**Figure 2A**). Furthermore, we also found a slight, although not significant, increase in P2Y_1_ expression on female platelets. Conversely, there was no difference in P2Y_12_, CD73, and COX1 expression between male and female rat platelets (**Figure 2**).

### Female Platelets Show Enhanced ATPase and ADPase Activity

**Figure 3:**
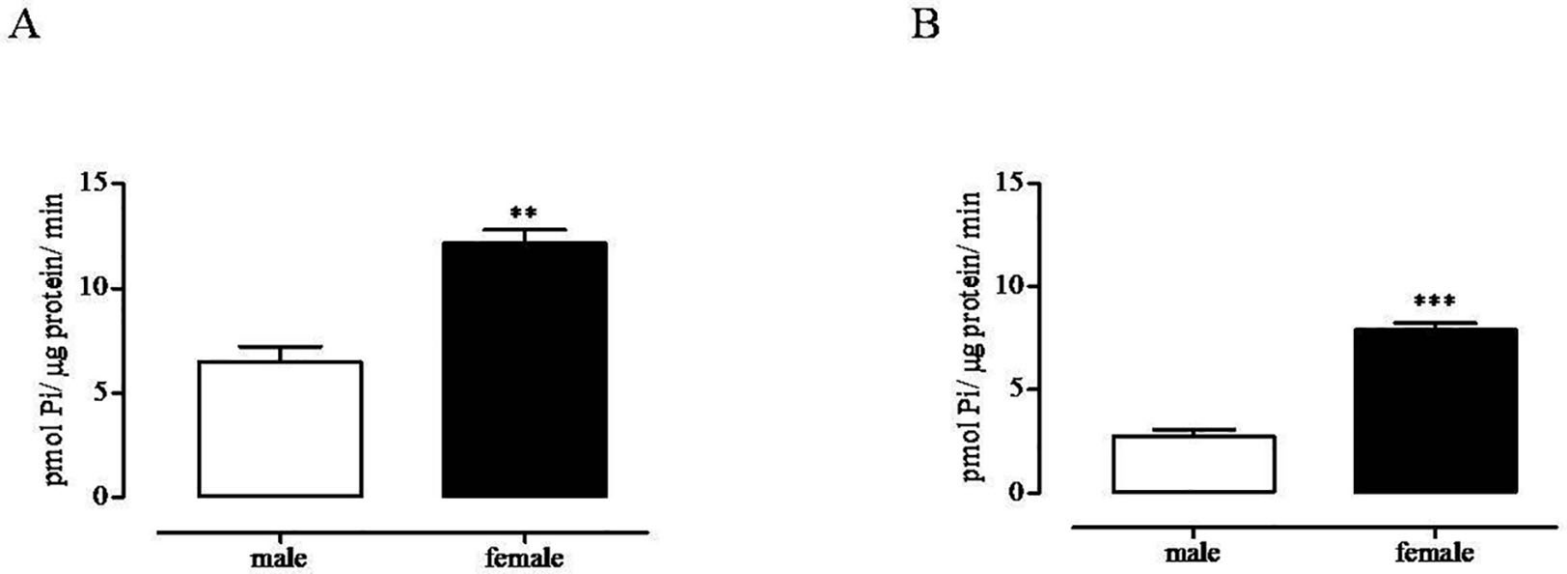
Sex difference in specific ATP **(A)** and ADP **(B)** hydrolysis mediated by CD39. ATPase and ADPase activity was evaluated on unstimulated platelet lysate from male (white bars) and female (black bars) rats by measuring inorganic phosphate (P*i*) released following incubation with ATP or ADP, respectively. For methodological details, see experimental section. **p < 0.01 and ***p < 0.001 *versus* male, two-tailed t test, N = 6.

Consistent with the increase in protein expression, platelet from female rats showed increased ATPase and ADPase activity compared to enzymatic activity on platelets from male rats (**Figure 3**).

### Clopidogrel Inhibits *Ex Vivo* Platelet Response to ADP But Does Not Affect CD39 Activity

**Figure 4:**
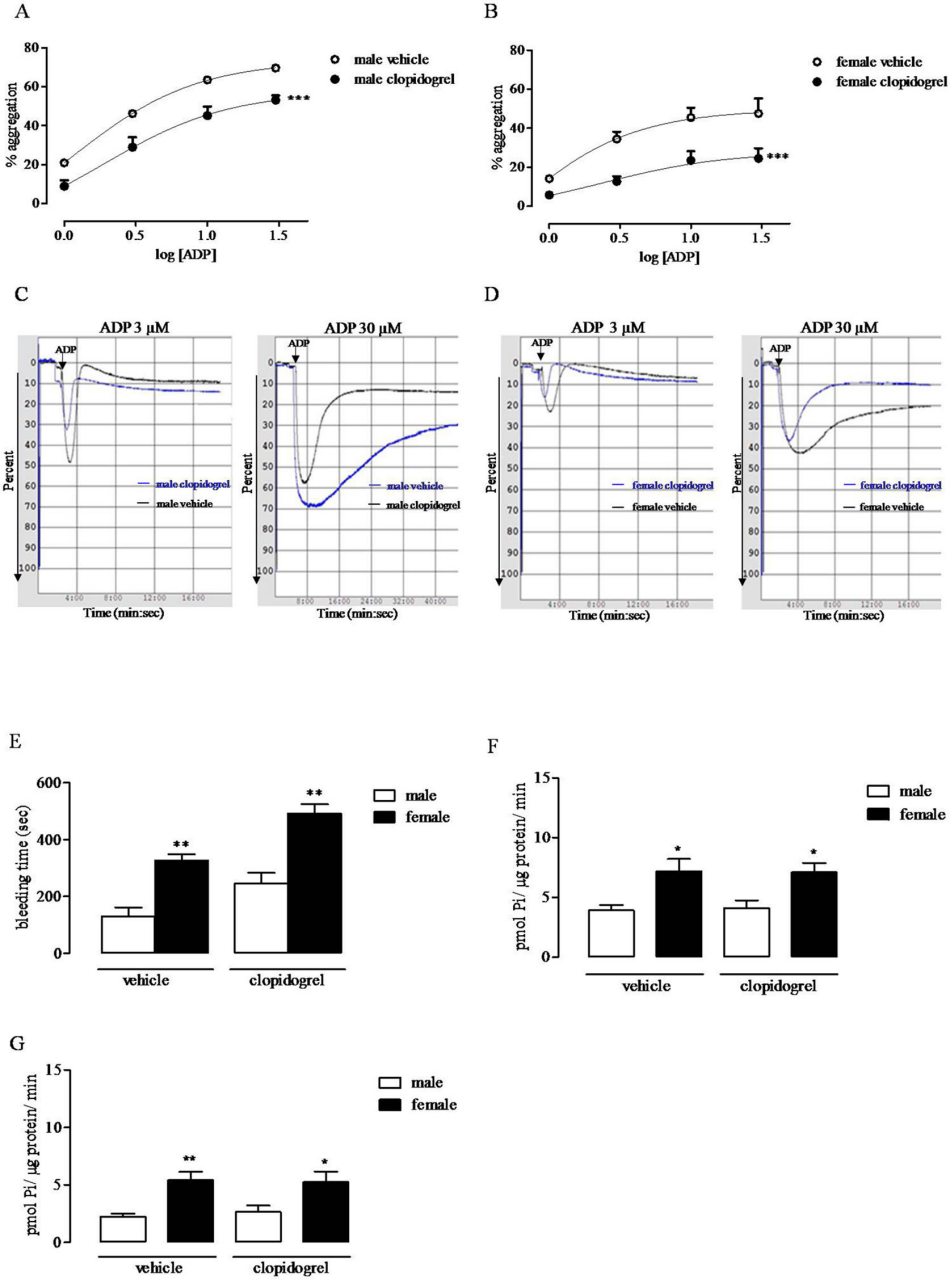
Ex vivo effect of treatment with clopidogrel on ADP-induced platelet aggregation, in male and female rats. Male **(A)** and female **(B)** rats were administered with clopidogrel (30 mg/kg, p.o.) or with the vehicle (0.5% sodium carboxymethyl cellulose, p.o.) and 2 h; thereafter, aggregation in response to ADP (1–30 μM) was evaluated. Aggregation was quantified and expressed as a percentage of maximum amplitude **(A, B)** ***p < 0.001, two-way ANOVA, N = 6. Typical records of platelet aggregation showing the ex vivo effect of clopidogrel are also reported **(C, D)**. Sex difference in bleeding time (seconds) evaluated 2 h after vehicle (0.5% sodium carboxymethyl cellulose, p.o.) or clopidogrel (30 mg/kg, p.o.) administration **(E)**. **p < 0.01 *versus* male, two-tailed t test, N = 5. ATPase **(F)** and ADPase **(G)** activity evaluated on platelet lysate from male and female rats administered with clopidogrel (30 mg/kg, p.o.) or with the vehicle (0.5% sodium carboxymethyl cellulose, p.o.) by measuring P*i* released following incubation with ATP or ADP, respectively. For methodological details, see experimental section. *p < 0.05 and **p < 0.01 *versus* male, two-tailed t test, N = 5.

Following *in vivo* treatment with clopidogrel, platelet aggregation in response to ADP was reduced both in female (Emax, from 49.72 ± 5.01% to 27.73 ± 5.3%, n = 5–6) and male (Emax, 73.27 ± 6.01% to 57.37 ± 4.73%; n = 5–6) rats (**Figure 4**). Rat treatment with clopidogrel did not affect CD39 activity.

### Female Rats Have Prolonged Bleeding Time

In the tail bleeding assay, blood loss was increased in female compared to male rats (female, 326 ± 21.39 s vs. male, 129 ± 32.52 s; p < 0.01, n = 5); this sex difference was observed also in clopidogrel-treated groups (female, 491.8 ± 33 s vs. male, 245 ± 38.14 s; p < 0.01, n = 5. **Figure 4E**).

## Discussion

Purine nucleotide and nucleoside hydrolysis, which is critical for vascular homeostasis, is mainly regulated by two ectonucleotidases, NTPDase-1/CD39 and ecto-5’-NT/CD73. CD39 is a key component of this pathway expressed on several cells, among which are platelets and endothelial cells, that hydrolyzes the proinflammatory and prothrombotic molecules ATP and ADP to AMP; thus, its activity has been shown to be of fundamental importance in maintaining an antithrombotic balance. The activity of CD73 controls extracellular adenosine levels which, in turn, by signaling through its receptors, exerts antithrombotic, anti-inflammatory and cardio protective effects (Koziak et al., 2008; Huttinger et al., 2012).

There is clinical evidence of gender difference in cardiovascular events; it has been shown that events prevail in men compared to women, and in particular the main cause of death is represented by coronary events; however, such a difference is age-dependent, since in the old women, cardiovascular events become more dramatic than those caused in men (Mahmood et al., 2014; Di Giosia et al., 2017).

Platelets play a major role in the control of vascular homeostasis and thrombosis and also represent key elements at the interface between inflammation and hemostasis. The increased platelet reactivity associated to several inflammatory conditions may explain the increased cardiovascular risk of patient suffering of chronic immune inflammatory disorders (Cicala and Cirino, 1998; Esmon and Esmon, 2011; Jenne et al., 2013). Here, we found that platelets from female rats showed reduced *in vitro* reactivity in response to ADP, compared with platelets from male rats. Both the % of maximum amplitude and the duration of platelet aggregation were reduced in female rats compared to the effect observed in platelets from male rats. To date, gender difference in platelet reactivity is still controversial and varies among species (Haque et al., 2001; Patti et al., 2014). In humans, some studies highlight increased platelet reactivity of women; however, other studies have not confirmed this result (Breet et al., 2011). A recent study that aimed to assess the relationship between vascular risk factors and gender in cerebral ischemia patients has found higher platelet reactivity in man than women (Wiśniewski et al., 2019). In rats, the increased platelet reactivity of male compared to female has been described since the 1970s (Johnson et al., 1975; Tantry et al., 2012). It has been suggested that sexual hormones might influence nucleotide hydrolysis, since platelets from ovariectomized rats show a decrease in ATP, ADP, and AMP hydrolysis (Pochman et al., 2005). Furthermore, recently, other experimental studies have outlined an effect of estrogen on purine metabolism (Mitrović et al., 2017; Grković et al., 2019; Shih et al., 2019). Here, we found increased expression of CD39 paralleled by increased ATPase and ADPase activity on female platelets, compared to the expression and activity evaluated on male platelets. There is much evidence that CD39 through its activity may have antiplatelet effects (Sévigny et al., 2002). In a murine model over-expressing CD39, reduced ex vivo platelet aggregation and resistance of thrombus formation have been demonstrated (Huttinger et al., 2012). Consistently, in a rat model of thrombosis, we have demonstrated a reduced CD39 activity and expression induced by IL-17, giving evidence for a role of CD39 in linking thrombosis and inflammation (Maione et al., 2014). Furthermore, there is evidence that natural products may have a protective cardiovascular effect by acting through the modulation of CD39 activity (Schmatz et al., 2013; Caiazzo et al., 2016). In view of these findings, it has been suggested that CD39 enzyme may offer promising opportunities in the management of vascular thromboinflammatory disorders. Thus, it is possible that an increased CD39 expression and activity contributes to the reduced response of female platelets to ADP, compared to the response of male platelets. This hypothesis is strengthened by evidence that when platelets from female rats were incubated with CD39 inhibitor, ARL67156, ADP-induced aggregation was strongly increased, while ARL67156 did not affect male platelet aggregation in response to ADP. Thus, the increased CD39 expression and activity on female platelets was functionally relevant compared to male platelets that were completely insensitive to the inhibition of CD39 enzyme activity.

ADP activates platelets through two G-protein coupled receptors, P2Y_1_ and P2Y_12_, which signal through Gq and Gi, respectively. P2Y_1_ receptor leads to shape change and reversible platelet aggregation, while P2Y_12_ activation leads to sustained, irreversible platelet aggregation. Both receptors are required for a physiological response to ADP (Burnstock and Williams, 2000; Hechler and Gachet, 2011). Clopidogrel is an extensively used antiplatelet drug to prevent thrombotic events (Lischke and Schneider, 2011). There are human studies describing higher on-treatment platelet reactivity of women compared to men (Berger et al., 2009; Breet et al., 2011); however, the cause of this finding has not yet established, neither the clinical implication. Here, we investigated platelet reactivity to ADP, following rat treatment with clopidogrel, in male and female comparatively. We found that rat treatment with clopidogrel inhibited *ex vivo* platelet aggregation in response to ADP, in both male and female rats. The effect was particularly evident at high ADP concentration, consistent with P2Y_12_ receptor inhibition; nonetheless, *in vivo* treatment with clopidogrel, did not abolish the difference in platelet reactivity in response to ADP between male and female rats, suggesting that treatment with clopidogrel inhibits *ex vivo* platelet aggregation regardless of basal platelet reactivity. Thus, it is possible that physiological mechanisms at the basis of the reduced platelet reactivity of female rats synergize with clopidogrel effect. Bleeding time is a test to assess platelet functionality; it is affected by changes in platelet reactivity and/or changes in molecules involved in platelet recruitment on vessels (Harrison and Lordkipanidzé, 2013). We found that bleeding time was prolonged in female rats compared to male rats. Even this result would be congruent with increased CD39 expression and activity on platelets from female rats. Indeed, there is evidence that increased CD39 activity also affects bleeding time and, in the last year, a great effort is being done to target CD39 antithrombotic and/or anti-inflammatory activity avoiding bleeding complications (Degen et al., 2017; Sashindranath et al., 2017; Granja et al., 2019). We also found that female platelets showed a slight increase in P2Y_1_ receptor expression compared to the expression found on male platelets. We do not know if such a difference may explain the different functionality we observed between male and female platelets; however, it has been described that the P2Y_1_ and P2Y_12_ receptors cross-communicate and, on one hand, P2Y_12_ receptor contributes to the P2Y_1_-mediated calcium response through phosphoinositolo-3 kinase activation but, on the other hand, P2Y_1_ negatively regulates P2Y_12_ activity (Hardy et al., 2004). Thus, it is possible that in platelets from female rats the balance between CD39 and P2Y receptor expression, which dictates ADP availability and signaling, respectively, leads platelets to a reduced response to ADP.

In conclusion, we found that female rats, in comparison to male rats, show reduced platelet reactivity in response to ADP, *in vitro*, and increased bleeding time that may be related to increased CD39 expression and activity on platelets. Consistently, platelets from female rats are sensitive to CD39 inhibition, while platelets from male rats are not.

Taken together, our data highlight the importance of CD39 in platelet function.

Although additional studies are required to predict gender difference in platelet reactivity, we suggest that the sex variability in platelet ATPase and ADPase activity should be taken into account for understanding more in depth the molecular mechanisms behind gender difference in cardiovascular risk.

## Data Availability Statement

The datasets generated for this study are available on request to the corresponding author.

## Ethics Statement

The animal study was reviewed and approved by the Italian Ministry of Health (459/2019-PR).

## Author Contributions

EC, RB, AR, AI, and CC contributed to the study design and data analysis. EC and RB performed the experiments and collected data. EC and CC wrote the manuscript. All authors reviewed and approved the manuscript.

## Funding

This work was supported by a grant from the Italian Ministry of University and Research (MIUR) PRIN 2017 (2017NKB2N4_003).

## Conflict of Interest

The authors declare that the research was conducted in the absence of any commercial or financial relationships that could be construed as a potential conflict of interest.
